# A Novel Low-Bit Quantization Strategy for Compressing Deep Neural Networks

**DOI:** 10.1155/2020/7839064

**Published:** 2020-02-18

**Authors:** Xin Long, XiangRong Zeng, Zongcheng Ben, Dianle Zhou, Maojun Zhang

**Affiliations:** ^1^College of Systems Engineering, National University of Defense Technology, Changsha 410073, China; ^2^College of Computer, National University of Defense Technology, Changsha 410073, China; ^3^College of Intelligent Science, National University of Defense Technology, Changsha 410073, China

## Abstract

The increase in sophistication of neural network models in recent years has exponentially expanded memory consumption and computational cost, thereby hindering their applications on ASIC, FPGA, and other mobile devices. Therefore, compressing and accelerating the neural networks are necessary. In this study, we introduce a novel strategy to train low-bit networks with weights and activations quantized by several bits and address two corresponding fundamental issues. One is to approximate activations through low-bit discretization for decreasing network computational cost and dot-product memory. The other is to specify weight quantization and update mechanism for discrete weights to avoid gradient mismatch. With quantized low-bit weights and activations, the costly full-precision operation will be replaced by shift operation. We evaluate the proposed method on common datasets, and results show that this method can dramatically compress the neural network with slight accuracy loss.

## 1. Introduction

Deep neural networks, such as handwritten character, image recognition, and many burgeoning AI applications, have achieved great success in recent years [1–3]. All these achievements rely on complex deep models. In the 2012 ILSVRC contest, Krizhevsky constructed a multilayer network [4] with 60 million parameters, and this network has exceeded all previous methods in terms of classification accuracy. However, training the entire network requires 2 to 3 days. Deep networks introduce a large number of layers due to their complicated structure, thereby increasing the model size (such as 50, 200, 250, and 500 MB for GoogleNet, ResNet-101, AlexNet, and VGG-Net, respectively) [5], computational complexity, and demand for energy consumption. Therefore, embedding these properties onto mobile devices is a large challenge. In deep neural networks, the computational cost and memory consumption are mainly dominated by convolution operation, which is exactly the dot-product between weight and activation vector. Most existing techniques focus on weight sharing, pruning, quantization, and activations discretion [6–8]. They also exhibit large accuracy drop and high computation during training and testing with float operation. In this work, we introduce a method to train low-bit networks. On one hand, this study approximates activations through low-bit discretization. On the other hand, weight quantization and special update mechanism for discrete weights are introduced. With quantized low-bit network weights and output activations, the costly full-precision convolutional operation will be replaced by shift operation, and marginal accuracy cost will decrease slightly. Our method will be important on embedded devices, such as ASIC or FPGA for AI.

## 2. Related Work

In this section, we discuss related work from following aspects:

- *Pruning and Sharing*. Parameter pruning and sharing has been used both to reduce the complexity of neural network and to avoid model overfitting. [6, 9–11] propose method to find and prune the redundant connections with small weight values, quantize the weights via weight sharing. The run-time memory saving and compression effect are very limited by those simple methods.
- *Structured Pruning and Sparsifying*. Generally speaking, L1 norm, L2 norm, Group Lasso, and other regularization terms are efficient ways to learning sparse weight structures in numerous researches. Wen et al. [12] proposes Structured Sparsity Learning by using Group Lasso to sparsify multiple DNN structures (filters, channels, and even layers). Besides, the authors of [13–16] also try to train network with sparsity regularizer, and transform the problem of measuring channel importance into optimization problem.
- *Special Neural Architecture*. Reducing calculation FLOPs and accelerating inference process of neural networks by designing special architecture. Related researches including Mobile-Net [17, 18], Squeeze-Net [19], and Shuffle-Net [20] by adopting convolutional filters of small size, depth-wise convolution operations.
- *Weight and Activation Quantization*. Our proposed quantization method also falls into this category. Low-bit quantization methods mean that the network weights and activations are expressed by discrete values according to special mathematical method, which could replace the costly original floating-point operations by only accumulations or even binary logic operations. The authors of [21, 22] firstly constrain the weights to the binary and ternary space. It follows that both weights and activations are mapped into binary space or ternary space, i.e., binary neural networks (BNN) [7], XNOR-Net [8], and ternary neural networks (TNN) [23], which directly replace multiply-accumulate operations by logic operations. DoReFa-Net [24] not only quantizes weights and activations, but also quantizes gradients to low-bit width floating-point numbers with discrete states in the backward propagation.

## 3. Low-Bit Neural Networks

In this section, we concentrate on training quantized low-bit networks. Specifically, the activations of layer output are quantized by either zero or powers of two to reduce storage and calculations. The weights of network are also restricted in the same way to obtain a sparse model. By constraining weights and activations to zero or powers of two, the costly floating-point multiplication operation can be replaced by cheaper shift operations [13].

### 3.1. Dot-product Function

**Figure 1:**
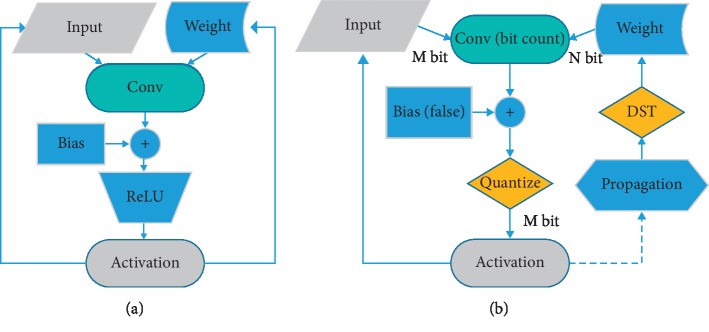
Convolution operation pipeline. (a) General convolution operation without quantization of weight and activation. (b) Description of proposed method with weight and activation quantized by low-bit.

Deep neural networks generally consist of multiple layers, and each neuron in different layers computes activation function:

(1) $$\begin{array}{c} z=f\left(w^{T}x+b\right), \end{array}$$

where *z* is output activation, *x* is input vector, *x* is weight vector, *b* is bias, and *f* is a nonlinear function, such as ReLU. Given the convolutional networks, the computational complexity is mainly dominated by the convolution operation. The key point of quantization for compression on hardware applications can be summarized into two aspects. One is the large memory required to store weights and activations. The other is the computational cost required to compute large numbers of dot-products. The difficulty lies in floating-point arithmetic, which is limited in practical applications [5] and is examined in this study.  Figure 1 shows the schematic of standard convolution process and our method (DST will be introduced in Section 3.3).

### 3.2. Low-Bit Activation Approximation

In this section, we have proposed a novel approximation strategy for activations quantization and corresponding suitable methods to keep the efficiency of backpropagation.

#### 3.2.1. Forward Approximation Process

In accordance with the discussion above, the activations of network are quantized by either zero or powers of two in this section. The optimization model is formulated as follows:

(2) $$\begin{array}{c} \left\{\begin{array}{c} q_{i+1}=2q_{i}\left(q_{i}\geq 0\right),\\ P\left(x\right)=\left\{\begin{array}{cc} q_{i}, & 0\leq x\in \left(t_{i},\;t_{i+1}\right],\\ -q_{i}, & 0\geq x\in \left[-t_{i+1},\;-t_{i}\right), \end{array}\right. \end{array}\right. \end{array}$$

where numerous parameter values within the interval (*t*_*i*_,  *t*_*i*+1_] ([−*t*_*i*+1_,   − *t*_*i*_)) are quantized into a common value *q*_*i*_(−*q*_*i*_), and *P*(*x*) is our new defined discrete activation function. We attempt to find the mean-squared error of all values for obtaining the optimal quantization method. Thus, optimization model (2) could be transformed into the following model:

(3) $$\begin{array}{c} \left\{\begin{array}{c} q_{i+1}=2q_{i},\\ P^{*}\left(x\right)=\underset{P}{\arg\min}\int^{}\varphi \left(x\right){\left(P\left(x\right)-x\right)}^{2}\text{d}x, \end{array}\right. \end{array}$$

where *φ*(*x*) is the probability density function of *x*. Following Cai's implementation [4], we apply batch normalization to the dot-product in (1) to determine the closeness of distributions to Gaussian with zero mean and unit variance. Accordingly, the optimal solution of (3) can be acquired by Lloyd's algorithm [25]. As a result, the best partition is

(4) $$\begin{array}{c} \left\{\begin{array}{c} P_{1}=\left\{x,\;0\leq x\leq x_{1}\right\},\\ P_{2}=\left\{x,\;x_{1}<x\leq x_{2}\right\},\\ \cdots \\ P_{v-1}=\left\{x,\;x_{v-2}<x\leq x_{v-1}\right\},\\ P_{v}=\left\{x,\;x_{v-1}<x\leq \infty \right\}, \end{array}\right. \end{array}$$

where *P*_*v*_ denotes different value interval of *x*. The endpoints of each interval are

(5) $$\begin{array}{c} \left\{\begin{array}{c} x_{1}=\frac{1}{2}\left(q_{1}+q_{2}\right),\\ x_{2}=\frac{1}{2}\left(q_{2}+q_{3}\right),\\ \cdots \\ x_{v-1}=\frac{1}{2}\left(q_{v-1}+q_{v}\right),\\ x_{v}=\frac{1}{2}\left(q_{v}+q_{v+1}\right), \end{array}\right. \end{array}$$

where we set up *q*_1_=0 and consider the symmetry of interval for *x* < 0. Therefore, the final optimization function of our quantizer is

(6) $$\begin{array}{c} \underset{q_{2}}{\arg\min}\left\{\int_{0}^{q_{2}/2}\varphi \left(x\right)x^{2}\text{d}x+\int_{q_{2}/2}^{3q_{2}/2}\varphi \left(x\right){\left(q_{2}-x\right)}^{2}\text{d}x+\overset{n}{\underset{i=3}{\sum }}\int_{\left(3/2\right)2^{i-3}q_{2}}^{\left(3/2\right)2^{i-2}q_{2}}\varphi \left(x\right){\left(2^{i-2}q_{2}-x\right)}^{2}\text{d}x+\int_{\left(3/2\right)2^{i-2}q_{2}}^{+\infty }\varphi \left(x\right){\left(2^{n-1}q_{2}-x\right)}^{2}\text{d}x\right\}, \end{array}$$

where *φ*(*x*) is the probability density function of standard normal distribution, *n* is the number of bits of activation function. Only one variable is considered in (6). Thus, the above-mentioned formula has a theoretical solution. However, we adopt the genetic algorithm in the experiments given the difficulty in solving segmented variable limit integral.  Table 1 shows the optimal error of different *q*_2_ values. With the further refinement of *q*_2_, we still obtain the same error value of 0.0189.

#### 3.2.2. Backward Approximation Process

Since dot-product values are equal within the same interval after using proposed forward approximation method, zero derivative almost everywhere. Thus, we have proposed a better possible backward solution here, and the final experimental results prove its feasibility in backpropagation process.

For 0 ≤ *x* ≤ *x*_1_, we approximate all values in this interval to be zero, similar to ReLU function, which does not need to update. Considering Gaussian distribution of dot-product mentioned above, plenty of activations fall into the interval near zero. We keep the gradient of this part as it was. For our quantization method, where activations are within interval, *P*_*v*_ has tiny probability. In this case, we need to limit their updates, preventing them from updating to other intervals and keep network accuracy. The derivative of quantization function has the following form:

(7) $$\begin{array}{c} \frac{\partial C}{\partial P}=\left\{\begin{array}{cc} 0, & 0\leq x\leq x_{1},\\ 1, & x\in \left(x_{1},x_{v-1}\right],\\ \frac{1}{\left(x-\left(x_{v-1}-1\right)\right)}, & x>x_{v-1}. \end{array}\right. \end{array}$$

For *x* < 0, consider interval symmetry. In our final experiment, we find this method keeping the efficiency of backpropagation and making learning stable.

### 3.3. Low-Bit Weight Quantization

The weight quantization shown above can be solved using various methods, such as BWN, DoReFa-Net, and XNOR [8, 21, 24]. However, we have to save full-precision weights in backward computation in these networks; this approach may cause frequent data exchange between external memory and parameter storage [26]. In this section, we propose a simple discretization function that maps weights into either zero or powers of two. This way replaces the floating-point operation by shift operations on hardware in backward process and avoids large computation and memory in hardware deployment.

#### 3.3.1. Weight Quantization in Forward Process

At the outset, we have considered weight discretization in forward process and updated them in constrained discrete domain. However, the weight is quantized into a discrete sequence of equal ratios here, which is difficult to update for the corresponding prescribed quantized values in backpropagation. The nonuniform distribution of discrete values is the main problem. Similar works such as BWN, DoReFa-Net, and XNOR, the derivative of weight in those ways is zero almost everywhere, making it apparently incompatible with backpropagation, and the gradient computation is based on the stored full-precision weights, and frequent data exchange is required during the training phase. In view of this, we seek to directly discrete network weights to either zero or powers of two in the backward process to avoid gradient mismatch problem, other than forward process.

#### 3.3.2. Weight Quantization in Backward Process

We introduce a weight update mechanism for discrete values in the backward process to avoid gradient mismatch. From previous works, we find that the weight value can be constrained to [−1, 1] in our quantization method. At the beginning, we introduce discrete state transformation (DST) problem for later use. We let Δ*w* be the variation in weight, *w* be the updated weight, and *w*′ be the raw weight. Thus,

(8) $$\begin{array}{c} \Delta w=w-w^{\prime }. \end{array}$$

**Figure 2:**
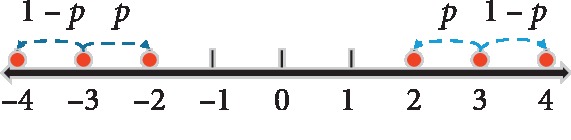
Binominal choice of undefined states for *w*_state_=±3.

*L* is the minimum interval of defined quantization weight, for (0, ±2^−2^, ±2^−1^, ±2^0^), and *L* is 2^−2^. For convenience, seven possible integer states (0, ±1, ±2, ±4) are considered when we constrain weight to (0, ±2^−2^, ±2^−1^, ±2^0^). Continuous weights need to be mapped into these discrete integer states. Accordingly, we adopt round operation:

(9) $$\begin{array}{c} w\text{\_state}=\text{round}\left(\frac{x}{2^{-2}}\right), \end{array}$$

where round is round operation in math and *x* is the arbitrary value within [−1, 1]. *w*_state_=±3 is not the defined discrete weight stated above. Thus, we introduce the binomial distribution to jump into the defined integer state on both sides:

(10) $$\begin{array}{c} w_{\text{state}}^{\prime }=p\times \left(\pm 2^{1}\right)+\left(1-p\right)\times \left(\pm 2^{2}\right), \end{array}$$

where the positive and negative signs are both positive or both negative at the same time, and *p* has a probability of 0 or 1 (we use random number for *p*, which has equal probability to be 0 or 1).  Figure 2 shows the above-mentioned process.

Finally, the weight state needs to be transformed into defined weight value:

(11) $$\begin{array}{c} w=w\text{\_state}\times 2^{-2}. \end{array}$$

In this way, we can transform continuous weights into defined discrete weights successfully. We transform the weight variation into defined discrete state transition. First, we decompose Δ*w* into integer and decimal parts by the minimum interval of quantization weight:

(12) $$\begin{array}{c} k=\text{sign}\left(\Delta w\right)\times \text{floor}\left(\frac{\left|\Delta w\right|}{L}\right),\\ v=\Delta w-k\times L, \end{array}$$

where floor represents the round down, *k* is the integer number of weight state transition, and *v* is the fine tuning parameter of weight state. Thus, the final state transition number is

(13) $$\begin{array}{c} \Delta w^{\prime }=\left(\text{sign}\left(\Delta w\right)\times \text{gate}+k\right)\times L, \end{array}$$

where gate submits to submits to binomial distribution, which has the opportunity *p*_1_ to be 1 and opportunity 1 − *p*_1_ to be 0. *p*_1_ is defined by fine tuning parameter *v*,

(14) $$\begin{array}{c} p_{1}=\tanh\left(\text{th}\times \left|v/L\right|\right). \end{array}$$

where th is a positive constant to adjust the state fine tuning probability *p*_1_, which will be explored in the experiments. Finally, we use the DST function, which is introduced above, to obtain the final quantized weight:

(15) $$\begin{array}{c} w\text{\_new}=\text{DST}\left(w^{\prime }+\Delta w^{\prime }\right). \end{array}$$

In this way, we constrain all weights to (0, ±2^−2^, ±2^−1^, ±2^0^). For other values, the same theory as described above applies.

## 4. Results and Discussion

In this section, we evaluate our proposed algorithm on MNIST (LeNet5), SVHN (VGG), and CIFAR10 (ResNet-18) for image classification by Pytorch. Most previous works do not quantize the first and last layers. In our method, we do not quantize the first layer only. Furthermore, we report the averaged results over three runs for each experiment by Adaptive Moment Estimation optimizer (ADAM).

### 4.1. Exploration the Quantization Combination of Weights and Activations

**Figure 3:**
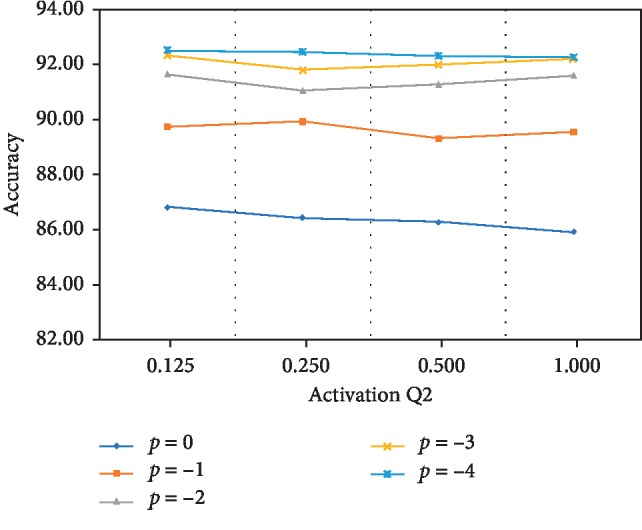
Comparison of accuracy with different combinations of quantized weights and activations. The horizontal axis shows the activation approximation bits and the vertical axis represents the quantization bits of network weight.

We illustrate the behavior of the different combinations of weights and activations with a standard ResNet-18 on the CIFAR10 dataset. We quantize all weights into (0, ±2^0^), (0, ±2^−1^, ±2^0^), and (0, ±2^−2^, ±2^−1^, ±2^0^). For the activation approximation, we use *q*_2_=0.125,  0.25,  0.5 *and* 1 as shown in Figure 1. For convenience, we set [*p*, *q*_2_] to define the quantization combination mode, where *p*=−2 represents above (0, ±2^−2^, ±2^−1^, ±2^0^), and the value of *q*_2_ determines the activation approximation degree. After cross combination, we set th=0.5 here, and the results are shown in Figure 3.

In general, weight quantization causes some accuracy degradation. Figure 3 confirms that accuracy increases with the deep degree of weight quantization. However, different approximation methods for activation do not influence test accuracy dramatically, but fluctuation during training occurs. Our method is also evaluated on other datasets.  Table 2 shows the comparison results under same conditions and the results from [27]. As elaborated above, BWN, TWN, and XNOR methods quantize weights to 1 or 2 bits of floating point every layer but not in the entire network. However, our method achieves 2 or 3 bits of fixed-point in the entire network and can be used with shift operation on ASIC or FPGA. To demonstrate the effectiveness of proposed method, we also show the comparison results on CIFAR100 with more complex model (ResNet-34, ResNet-50), as shown in Table 3.

### 4.2. Effect with the Change of th

**Figure 4:**
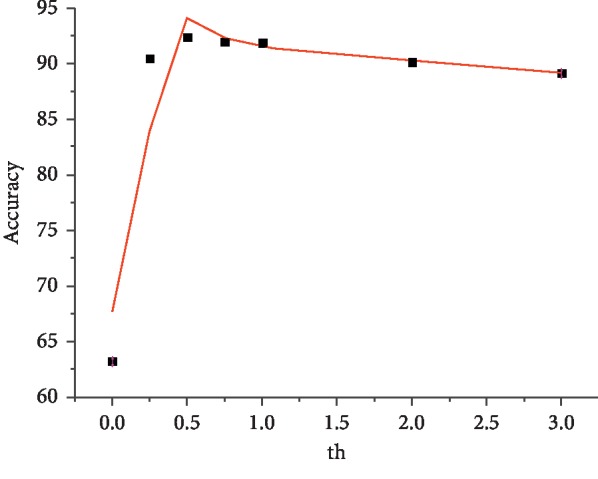
Comparison of accuracy with different combinations of quantized weights and activations. The horizontal axis shows the activation approximation bits and the vertical axis represents the quantization bits of network weight.

We explore the effect of parameter th in this section. As explained above, th adjusts the weight state fine tuning probability to influence the final learning accuracy.  Figure 4 shows the results, which indicate excellent nonlinearity. Here, we test the combination [−3, 0.125]. Evidently, the curve has the best accuracy at approximately th=0.5, whereas larger or smaller values may result in slight improvement. The same result is obtained for other combinations after several experiments. Thus, we adopt th=0.5 for all experiments in this study.

### 4.3. Influence of the First and Last Layer Quantization

The first and last layers are critical to network quantization research according to previous works. In the current study, all our experiments do not quantize the first layer only. We attempt to investigate the influence of first layer quantization. The results are summarized in Table 4. We test the weight and activation quantization combination [−3, 0.125] here. “+” and “−” indicate with or without weight quantization of the corresponding layer.

Evidently, accuracy degradation may occur when quantizing the first or last layer. Our method is slightly better than BNN but is not better than BWN which quantizes weights only.

### 4.4. Parameter Sparsity

Most of the current AI applications are based on ResNet. Thus, we analyze parameter sparsity on ResNet-18. Previous methods clip a large number of weights by setting most weights of small values to zero but not to be exactly zero [28]. By contrast, our method can obtain precise zero value weights. The results of our method using the combination [−3, 0.125] are shown in Table 5.

Evidently, our method can obtain large sparsity on convolutional layer parameters, and several top layers of the network may be valuable for final evaluation. The back layer is sparser than the front one, which may be pruned in our future work. As an attempt, we prune the pretty sparse layers (conv19, conv20), finding accuracy dropping little and obtaining more compact layers. More meaningfully, training and inference time are reduced in a certain extent which may significant for hardware implementations.

## 5. Conclusions

In deep networks, computational cost and storage capacity are key factors that directly affect the learning performance. Compression and acceleration of networks aim to reduce the redundancy of complex models. Accordingly, we introduce a method to train networks with weights and activations quantized by several bits. We find that our method drops network accuracy slightly, whereas it decreases storage and computation sharply. Interestingly, our quantified model has evident sparsity, which may be pruned on ASIC or FPGA for AI in the future.

## Figures and Tables

**Table 1:** Expect error of different *q*_2_ values.

Scheme	*q*_2_ = 0.0625	*q*_2_ = 0.125	*q*_2_ = 0.25	*q*_2_ = 0.5	*q*_2_ = 1
*n*=3	0.4078	0.3298	0.2106	0.0825	0.0458
*n*=4	0.3298	0.2103	0.0795	0.0239	0.0443
*n*=5	0.2102	0.0791	0.0209	0.0223	0.0443
*n*=6	0.0790	0.0205	0.0193	0.0223	0.0443
*n*=7	0.0204	**0.0189**	0.0193	0.0223	0.0443
*n*=8	**0.0189**	**0.0189**	0.0193	0.0223	0.0443

**Table 2:** Test error comparison on multiple datasets.

Method	Weight (bit)	Activation (bit)	MNIST	SVHN	CIFAR10
BNN	1	1	1.27	2.53	8.46
BWN	1	32	0.54	—	7.25
TWN	2	32	0.65	—	7.44
DoReFa	8	8	—	2.30	—
Ours	3	3	0.96	2.14	7.48

**Table 3:** Accuracy comparison on CIFAR100.

avb	BNN	XNOR	Ours
ResNet-34	48.81/78.32	53.28/81.29	61.33/87.22
ResNet-50	52.07/81.60	59.20/85.32	62.92/88.65

**Table 4:** Accuracy comparison with quantization of first or last convolutional layer.

CIFAR10/MNIST	BWN	BNN	Ours
+ First − last	92.37/99.37	91.40/98.66	92.08/98.86
+ First + last	92.21/99.41	91.30/98.52	91.96/98.55
− First + last	92.52/99.38	91.47/98.71	92.52/98.75
− First − last	92.75/99.46	91.54/98.73	92.12/99.04

**Table 5:** Sparsity of ResNet-18 on CIFAR10.

Layers (weight tensors)	Full precision (1 − sparsity) (%)	Our method (1 − sparsity) (%)
Conv1 (64, 3, 3, 3)	100	100
Conv2 (64, 64, 3, 3)	100	85.32
Conv3 (64, 64, 3, 3)	100	86.71
Conv4 (64, 64, 3, 3)	100	85.84
Conv5 (64, 64, 3, 3)	100	85.10
Conv6 (128, 64, 3, 3)	100	86.04
Conv7 (128, 128, 3, 3)	100	83.46
Conv8 (128, 64, 1, 1)	100	86.52
Conv9 (128, 128, 3, 3)	100	82.88
Conv10 (128, 128, 3, 3)	100	80.75
Conv11 (256, 128, 3, 3)	100	77.45
Conv12 (256, 256, 3, 3)	100	70.23
Conv13 (256, 128, 1, 1)	100	77.74
Conv14 (256, 256, 3, 3)	100	59.51
Conv15 (256, 256, 3, 3)	100	42.64
Conv16 (512, 256, 3, 3)	100	22.16
Conv17 (512, 512, 3, 3)	100	10.72
Conv18 (512, 256, 1, 1)	100	41.56
Conv19 (512, 512, 3, 3)	100	5.02
Conv20 (512, 512, 3, 3)	100	3.46
1 − Sparsity	100	23.32
Accuracy	93.74	92.52

## Data Availability

The data used to support the findings of this study are open datasets which could be found in general websites, and the datasers are also freely available.
